# Ethnicity does not account for differences in the health-related quality of life of Turkish, Moroccan, and Moluccan elderly in the Netherlands

**DOI:** 10.1186/s12955-014-0138-8

**Published:** 2014-10-01

**Authors:** Ilona Verhagen, Wynand JG Ros, Bas Steunenberg, Niek J de Wit

**Affiliations:** Julius Center for Health Sciences and Primary Care, University Medical Center Utrecht, Mailbox 85500, 3508 GA Utrecht, the Netherlands

**Keywords:** Health-related quality of life, Elderly immigrants, Ethnicity, Multimorbidity, Loneliness

## Abstract

**Background:**

Data on how different groups of elderly immigrants perceive health-related quality of life (HRQOL) is scarce and research on the influence of ethnicity on HRQOL across ethnic groups is missing. Measuring HRQOL may help to detect cross-cultural differences and to decide whether ethnic-specific health and prevention programmes are required to improve HRQOL. We investigated differences in HRQOL among three elderly immigrant populations with a special focus on the contribution of ethnicity, in addition to other well-known determinants, to HRQOL.

**Methods:**

Data were collected between October 2011 and July 2012 as part of the project entitled "Stem van de oudere migrant", a quasi-experimental study in the Netherlands focussing on health of immigrant elderly. A survey was conducted among 201 elderly (aged 55 years and older) Moroccans (98), Turks (69), and Moluccans (34). HRQOL was assessed using the SF-12, measuring physical and mental health composite scores (PCS resp. MCS). Chi-square tests and ANOVAs were performed for group comparison. Hierarchical multiple linear regressions were conducted to examine whether ethnicity uniquely contributed to the observed variance in HRQOL when multimorbidity, loneliness, socio-demographics, and acculturation were taken into account.

**Results:**

Moroccans had the lowest scores on PCS (34.3 ± 31.4) and MCS (42.1 ± 27.0), followed by Turks (45.7 ± 27.0 for PCS and 54.7 ± 22.2 for MCS), and Moluccans (71.7 ± 21.2 for PCS and 74.4 ± 22.1 for MCS). Ethnicity was not independently associated with PCS and MCS scores, in contrast to loneliness (PCS β -0.461, *p* < 0.001 and MCS β -0.435, *p* < 0.001) and multimorbidity (PCS β -0.380, *p* < 0.001 and MCS β -0.398, *p* < 0.001). Gender was independently associated with PCS (β 0.148, *p* = 0.026) and attachment to Dutch culture with MCS (β 0.144, *p* = 0.029).

**Conclusions:**

The lower level of HRQOL reported by elderly immigrant populations was affected by multimorbidity and loneliness but not ethnicity. Similar to native elders, interventions aiming at improving HRQOL for immigrant elderly should focus on loneliness and (mental and physical) disease. Finally, health literacy deserves attention to maintain health.

**Trial registration:**

ISRCTN89447795

## Introduction

The population in western countries is ageing and becoming more ethnically diverse. The health status [1-5] and health-related quality of life (HRQOL) [1,6] of immigrant elderly is often less favourable compared to native elderly. So far, data on how different groups of elderly immigrants perceive HRQOL are scarce, and research on the influence of ethnicity on HRQOL across ethnic groups is lacking. Measuring HRQOL may help to detect cross-cultural differences and to determine the necessity for ethnicity-specific health and prevention programmes to improve HRQOL.

Optimal health for the elderly is not a goal, as such, but it enables this population to maintain their independence, mobility and participation in life activities, as well as to respond to the challenges of old age [7]. The consequences of impaired health, rather than the absence of disease, mainly determine the quality of life for the elderly. Therefore, HRQOL is the most adequate parameter of well-being in older populations.

Loneliness, chronic health conditions, and multiple morbidities are well-known determinants of HRQOL [8-10]. Gender has been reported as a factor determining the HRQOL of native and immigrant elders [10-12]. Other factors that have been associated with HRQOL in immigrant elderly are educational level and co-morbidity [1]. Differences in HRQOL between foreign- and native-born elderly suggest that ethnicity may be another factor associated with HRQOL.

In the present study, we assessed differences in HRQOL among different ethnic groups of immigrant elders and whether, in addition to established determinants of HRQOL, such as multimorbidity, loneliness, socio-demographics, and acculturation, ethnicity contributed independently to HRQOL.

## Methods

### Study population

The study population consisted of a sample of elderly Moroccans, Turks, and Moluccans who participated in the project entitled "Stem van de oudere migrant" [13]. This quasi-experimental study aimed to assess the effectiveness of community health workers (CHWs) in improving access to health care facilities. The design and aims of that study are described elsewhere [13].

Turks and Moroccans immigrated to the Netherlands in the 1960s and early 1970s in pursuit of work and, later on, for family reunification or family formation. Moluccans, former soldiers in the Dutch colonial army, and their families were transferred to the Netherlands in 1951 after the decolonisation of Indonesia and were housed in resettlement camps in remote areas. Also included in the study were descendants of Moluccan immigrants who were born between 1951 and 1957 and raised in the Netherlands in the relatively isolated camps where the living situation, language and customs resembled those of the country of origin. Eligible participants were aged 55 years or older, living independently and currently not being treated for severe psychiatric disorders. Participants were recruited through CHWs. Details of the recruitment procedures have been described elsewhere [13].

### Data collection

Data were collected between October 2011 and July 2012 by means of a structured personal interview conducted at home or at another location chosen by the participant. Trained interviewers from similar ethnic backgrounds verbally administered all questionnaire items by reading aloud the questions and the response options in the language of the participant. Standardised, translated versions of the questionnaire were used to minimise the variation in results and ensure the compatibility of interviews across all interviewers.

### Ethical principles

All the participants provided oral or written informed consent. The Medical Ethics Committee of the University Medical Center Utrecht (UMCU) did not consider the study to be subject to the Medical Research Involving Human Subjects Act (WMO in Dutch); therefore, full ethical assessment was not required. The study is registered with the ISRCTN register: ISRCTN89447795.

### Outcome, determinants, and measurements

The primary outcome was self-reported HRQOL, measured with the Short Form-12 (SF-12) [14]. The SF-12 is a widely used instrument to measure physical and mental aspects of HRQOL. The SF-12 is comprised of 12 items and yields 8 domain scores Physical Functioning; Role Physical, Bodily Pain, General Health, Vitality, Social Functioning, Role Emotional, and Mental Health that can be condensed into physical and mental health summary scores respectively the Physical Component Summary (PCS) and the Mental Component Summary (MCS).

We assessed the following determinants:

*Ethnicity* was determined by self-reported country of birth or birth in one of the Moluccan resettlement camps.

Regarding socio-demographic variables, *socio-economic status* (SES) was determined using the Netherlands Institute for Social Research (SCP) status scores that are based upon the social status of a particular postcode area [15]. The number of *years of education* was measured by the sum of years of education in the country of origin and in the Netherlands. *Age, gender, and marital status* were self-reported. The number of *years of residence* in the Netherlands was determined from the year of immigration to the Netherlands. *Language proficiency* was measured by self-reported difficulty in speaking Dutch. Acculturation was measured using the adapted Psychological Acculturation Scale (PAS) [16], originally developed by Tropp et al. [17]. The PAS consists of two scales evaluating emotional attachment to Dutch culture (D-PAS) and culture of origin (in this study defined as C-PAS). Multimorbidity was defined as the presence of two or more chronic conditions from a selection of 17 self-reported chronic conditions. *Loneliness* was measured by the De Jong Gierveld loneliness scale [18].

### Data analysis

We compared the three ethnic groups with respect to socio-demographic information, health characteristics, and HRQOL. Chi square tests were performed to evaluate differences in nominal and ordinal variables. Analysis of variance (ANOVA) was used for continuous variables with the Welch modification when the assumption of homogeneity of variance was not met. A significant ANOVA was followed by post hoc multiple comparison testing using Bonferroni’s test. The Games-Howell test was used when the assumption of homogeneity of variance was violated.

Psychometric properties of the translated instruments were absent for the immigrant groups in our study. We therefore assessed Cronbach’s alpha in the total population and for the three immigrant groups separately.

Hierarchical multiple regressions were executed for PCS and MCS to examine whether ethnicity contributed uniquely to the variance in HRQOL when multimorbidity, loneliness, socio-demographics, and acculturation were taken into account. Correlations lower than 0.80, suggesting that multicollinearity was at an acceptable level, permitted the entry of the variables [19]. Independent variables were entered into five blocks in the hierarchical multiple regression. In the first block, PCS was regressed on ethnicity only. In the second block, PCS was regressed on multimorbidity, in addition to ethnicity. In third block, loneliness was added. In the fourth block, acculturation, years of residence in the Netherlands, and language proficiency were entered. In the final block, age, gender, SES, years of education, and marital status were added. An identical regression analysis was performed with MCS as the dependent variable.

Statistical significance level was set at 0.05. All analyses were performed in SPSS 20.0 for Windows.

## Results

### Sample

A total of 478 elderly immigrants were approached for participation in the study, and 201 completed the questionnaire. The overall response rate was 42.1% (38.1% among Moroccans, 79.3% among Turks, and 25.4% among Moluccans).

As shown in Table 1, the mean (±SD) age of the Turks was 63.3 ± 5.0; of the Moroccans was 67.1 ± 7.3; and of the Moluccans was 69.7 ± 8.6. The average number of years of residence in the Netherlands was 59.4 ± 4.8 for the Moluccans, 34.7 ± 10.6 for the Moroccans, and 34.2 ± 8.1 for the Turks. A high proportion of Moroccans (100%) and Turks (89.9%) showed an SES below the Dutch average. Additionally, 76.8% of the Turks, 57.1% of the Moroccans, and 44.1% of the Moluccans reported multimorbidity. Moroccans scored highest on loneliness (7.3 ± 4.2), followed by Turks (5.4 ± 3.8), and Moluccans (1.6 ± 2.0).

**Table 1:** **Socio-demographic and health characteristics of the study sample**

	**Moroccan**	**Turkish**	**Moluccan**	***p*** **-value**
	**N = 98**	**N = 69**	**N = 34**	
Age (mean, SD)	67.1 (7.3)^1^	63.3 (5.0)^2^	69.7 (8.6)^3^	<0.001
Age (%)				0.001
55-59 years	18.4	27.5	14.7	
60-69 years	41.8	60.9	38.2	
≥70 years	39.8	11.6	47.1	
Gender (%)				0.724
Male	56.1	50.7	50.0	
Years of residence in the Netherlands^a^ (mean, SD)	34.7 (10.6)^4^	34.2 (8.1)^3^	59.4 (4.8)^2^	<0.001
Years of education (mean, SD)	1.1 (3.0)^2^	3.7 (3.2)^2^	10.4 (4.3)^2^	<0.001
SES below Dutch average^b^ (%)	100	89.9	55.9	<0.001
Marital status (%)				0.529
Married	82.3	88.4	82.4	
Divorced/widowed	17.7	11.6	17.6	
Acculturation (PAS)				
D-PAS^c^ (mean, SD)	19.3 (4.6)	18.8 (5.4)^3^	21.9 (6.2)^3^	0.048
C-PAS^d^ (mean, SD)	23.1 (5.1)^2^	26.9 (3.8)^1^	26.2 (3.8)^4^	<0.001
Language proficiency (mean, SD)^e^	2.7 (0.8)^2^	2.3 (0.5)^2^	3.6 (0.6)^2^	<0.001
Multimorbidity (%)				0.004
0 chronic conditions	20.4	10.1	38.2	
1 chronic condition	22.4	13.0	17.6	
≥2 chronic conditions	57.1	76.8	44.1	
Loneliness (De Jong Gierveld	7.3 (4.2)^2^	5.4 (3.8)^2^	1.6 (2.0)^2^	<0.001
loneliness scale)^f^ (mean, SD)				
HRQOL (SF-12)				
PCS^g^ (mean, SD)	34.3 (31.4)^2^	45.7 (27.0)^2^	71.7 (21.2)^2^	<0.001
MCS^h^ (mean, SD)	42.1 (27.0)^2^	54.7 (22.2)^2^	74.4 (22.1)^2^	<0.001

^a^Moluccans born in the Netherlands were not asked.

^b^Status scores were classified into SES above the Dutch average and below the Dutch average. The average status score in the Netherlands was used as cut-off point.

^c^Ranged from 6 to 30. A higher score means a greater sense of emotional attachment and belonging within the Dutch culture.

^d^Ranged from 6 to 30. A higher score means greater sense of emotional attachment and belonging within the culture of origin.

^e^Ranged from 1 to 4. A higher score means a better command of the Dutch language.

^f^Ranged from 0 to 11. A higher score indicates a greater experience of feelings of loneliness.

^g^Ranged from 0 to 100. A higher score indicates better physical health.

^h^Ranged from 0 to 100. A higher score indicates better mental health.

^1^Means of Moroccans and Turks differ significantly (*p* < 0.05).

^2^Means of Moroccans, Turks, and Moluccans differ significantly (*p* < 0.05).

^3^Means of Moluccans and Turks differ significantly (*p* < 0.05).

^4^Means of Moluccans and Moroccans differ significantly (*p* < 0.05).

### Psychometric properties

The overall Cronbach’s alpha α was 0.89 for PCS (0.80 for Moroccans, 0.82 for Turks, and 0.75 for Moluccans) and 0.82 for MCS (0.90 for Moroccans, 0.88 for Turks, and 0.77 for Moluccans). Overall Cronbach’s alpha α was 0.79 for the D-PAS (0.86 for Moroccans, 0.75 for Turks, and 0.85 for Moluccans) and 0.85 for the C-PAS (0.91 for Moroccans, 0.70 for Turks, and 0.70 for Moluccans). Cronbach’s alpha α was 0.92 for the loneliness scale (0.93 for Moroccans, 0.91 for Turks, and 0.69 for Moluccans).

### Sample characteristics

Table 1 shows that differences among the ethnic groups were found in the categories of years of education (*p* < 0.05), language proficiency (*p* < 0.05), loneliness (*p* < 0.05), and HRQOL (*p* < 0.05). Regarding HRQOL, Moroccans had the lowest scores on PCS (34.3 ± 31.4) and MCS (42.1 ± 27.0), followed by Turks (45.7 ± 27.0 for PCS and 54.7 ± 22.2 for MCS), and Moluccans (71.7 ± 21.2 for PCS and 74.4 ± 22.1 for MCS).

### Determinants of HRQOL

Multiple regression analysis (Table 2, Model 5) showed that ethnicity was not independently associated with PCS and MCS, in contrast to loneliness (PCS β -0.461, *p* < 0.001 and MCS β -0.435, *p* < 0.001) and multimorbidity (PCS β -0.380, *p* < 0.001 and MCS β -0.398, *p* < 0.001). Gender was independently associated with PCS (β 0.148, *p* = 0.026) and attachment to Dutch culture with MCS (β 0.144, *p* = 0.029).

**Table 2:** **PCS and MCS assessed by linear regression analysis (β’s)**

**Physical Component Summary (PCS)**	**Model 1**		**Model 2**		**Model 3**		**Model 4**		**Model 5**	
	**R** ^**2**^ **adj. 0.114**		**R** ^**2**^ **adj. 0.335**		**R** ^**2**^ **adj. 0.506**		**R** ^**2**^ **adj. 0.516**		**R** ^**2**^ **adj. 0.538**	
	***F*** **11.624 (** ***p*** **< 0.001)**		***F*** **28.711 (** ***p*** **< 0.001)**		***F*** **43.286 (** ***p*** **< 0.001)**		***F*** **23.027 (** ***p*** **< 0.001)**		***F*** **15.775 (** ***p*** **< 0.001)**	
	**β**	***p*** **-value**	**β**	***p*** **-value**	**β**	***p*** **-value**	**Β**	***p-*** **value**	**β**	***p*** **-value**
Ethnic group (vs. Moluccans)										
Turkish	−0.396	0.001	−0.321	0.001	−0.140	0.115	0.040	0.731	0.033	0.803
Moroccan	−0.546	<0.001	−0.564	<0.001	−0.251	0.009	−0.093	0.430	−0.050	0.735
Multimorbidity (vs. no multimorbidity)			−0.480	<0.001	−0.395	<0.001	−0.395	<0.001	−0.380	<0.001
Loneliness					−0.469	<0.001	−0.454	<0.001	−0.461	<0.001
Years of residence in the Netherlands							0.132	0.081	0.088	0.359
Dutch-PAS							0.068	0.291	0.033	0.616
Country of origin-PAS							0.005	0.935	0.010	0.871
Language proficiency							0.047	0.483	0.022	0.740
Age									−0.042	0.553
Male (vs. female)									0.148	0.026
SES below Dutch average (vs. above Dutch average)									−0.022	0.712
Education years									0.119	0.130
Married (vs. divorced/widowed)									−0.102	0.081
**Mental Component Summary (MCS)**	**Model 1**		**Model 2**		**Model 3**		**Model 4**		**Model 5**	
	**R** ^**2**^ **adj. 0.117**		**R** ^**2**^ **adj. 0.319**		**R** ^**2**^ **adj. 0.480**		**R** ^**2**^ **adj. 0.527**		**R** ^**2**^ **adj. 0.529**	
	***F*** **11.891 (** ***p*** **< 0.001)**		***F*** **26.767 (** ***p*** **< 0.001)**		***F*** **39.085 (** ***p*** **< 0.001)**		***F*** **23.955 (** ***p*** **< 0.001)**		***F*** **15.233 (** ***p*** **< 0.001)**	
	**β**	***p-*** **value**	**β**	***p*** **-value**	**β**	***p*** **-value**	**Β**	***p*** **-value**	**β**	***p*** **-value**
Ethnic group (vs. Moluccans)										
Turkish	−0.363	0.002	−0.319	0.002	−0.149	0.112	0.092	0.435	0.005	0.970
Moroccan	−0.560	<0.001	−0.606	<0.001	−0.304	0.003	−0.112	0.343	−0.240	0.114
Multimorbidity (vs. no multimorbidity)			−0.460	<0.001	−0.369	<0.001	−0.376	<0.001	−0.398	<0.001
Loneliness					−0.453	<0.001	−0.425	<0.001	−0.435	<0.001
Years of residence in the Netherlands							0.118	0.107	0.018	0.853
Dutch-PAS							0.156	0.014	0.144	0.029
Country of origin-PAS							0.008	0.902	0.009	0.881
Language proficiency							0.104	0.113	0.121	0.070
Age									0.056	0.439
Male (vs. female)									0.124	0.068
SES below Dutch average (vs. above Dutch average)									0.071	0.236
Education years									0.003	0.971
Married (vs. divorced/widowed)									−0.035	0.556

## Discussion

The present study revealed that elderly immigrant populations in the Netherlands experience different levels of HRQOL, but these differences are not related to ethnic background. Multimorbidity and loneliness counted for most of the differences in HRQOL. In addition, gender and attachment to Dutch culture moderately contributed to the variance in HRQOL. These outcomes suggest that elderly from different ethnic backgrounds have a more or less comparable level of HRQOL as long as their health status, social resources, and level of acculturation are comparable.

Within the different ethnic groups, Moluccans had, by far, the best HRQOL, whereas Moroccans had the poorest. The Turks scored in between these two groups. This trend was evident in both the physical and mental aspects of quality of life. In addition to our findings that multimorbidity and loneliness are important predictive factors, we performed additional analyses for the mean scores of HRQOL adjusted for these two factors. After adjustment, average physical and mental quality of life improved slightly for Moroccans and Turks, but not for Moluccans (adjusted mean PCS scores: Moroccans 38.8, Turks 47.7, Moluccans 53.0; adjusted mean MCS scores: Moroccans 45.7, Turks 55.2, Moluccans 59.5). The Moroccans, in contrast to the Turks, even after adjustment still showed significantly worse HRQOL than the Moluccans.

Our results raise the question: Does the mean score of older immigrants differ from that of the native Dutch older population? Comparison with available data on the average PCS and MCS scores for elderly from the native Dutch population in 2012 [20] showed that Moroccans and to a lesser extent Turks had a worse score on PCS than the Dutch (55–65 years 48.6; 65–75 years 47.4; ≥75 years 42.4). The Moluccans had a better physical and mental quality of life. Moroccans had a worse score on MCS than the Dutch (55–65 years 53.5; 65–75 years 55.0; ≥75 years 54.0), Turks had a quite comparable score, and Moluccans had a better one. In general, with the exception of the Moluccans, we can conclude that HRQOL, particularly physical quality of health was poorer than the native Dutch elderly population.

Our results did not completely resemble earlier research. In line with Schellingerhout [1], Moluccans reported the best mental quality of life. Schellingerhout [1] and Denktaş [2] found no differences between Turks and Moroccans. However, in our study, Turks and Moroccans experienced different levels of HRQOL with Moroccans being the most disadvantaged. Differences in area of residence could explain discrepancies in HRQOL. The Turks in our study lived in a semi-urban setting. However, the Moroccans in our study and the Moroccans and Turks in the studies by Schellingerhout and Denktaş lived in deprived neighbourhoods in a large city. The literature suggests that poorer health status and harmful health behaviour occur more often in deprived neighbourhoods [21].

That multimorbidity as an important predictive factor for low HRQOL is consistent with research showing that chronic conditions and multiple morbidities are associated to poorer HRQOL [9,10]. Our study confirms this for elderly immigrants. Our results also confirm that loneliness is highly associated with low HRQOL in the elderly [8,22], but the causal relation can be interpreted in two different ways. On the one hand, a low HRQOL may lead to loss of social interactions and, ultimately, to feelings of loneliness [11]. On the other hand, loneliness may lead to experiencing poor HRQOL.

We found gender to be associated with physical quality of life, which is consistent with previous studies showing a better HRQOL for men, regardless of whether they were natives or immigrants [10-12]. As we corrected for age and multimorbidity these factors are not a plausible explanation. The association is more likely to be explained by the fact that women have a more acute perception of their health problems and consequently they are more likely to report them [12].

Our finding that attachment to Dutch culture is associated with a higher mental quality of life is in contrast to earlier research. Schellingerhout found no influence of acculturation [1]. This discrepancy might be due to differences in the acculturation measures used in the different studies. Schellingerhout used variables such as Dutch language use, informal contacts with native Dutch, and attitudes on care and family values as indicators of acculturation, whereas we focussed on individuals’ psychological identification with Dutch culture and the subjects’ cultures of origin. Nevertheless, it is quite understandable that elderly who are more emotionally attached to and integrated with Dutch people and culture perceive a better quality of life.

There are some limitations to our study that need to be considered when interpreting our results. First, although the SF-36, the parent questionnaire for the SF-12, and the SF-12 itself are widely used valid instruments that have been previously used to measure HRQOL including immigrants [1,2,6,12,23], measurement bias might have been occurred by the blending of health and physical and mental function concepts in the SF-36 [24,25]. As our study focused on the physical and mental health summary scores, it could still be possible that ethnicity affected HRQOL independently of physical and mental functioning. To exclude this possibility, additional analyses were performed on the SF-12 general health rating item (“In general how would you rate your health?”), which is in contrary to the other SF-12 items not specifically related to physical and mental function. It is one of the most frequently asked questions to assess health status. In line with our findings on the PCS and MCS, ethnicity was not associated with this single SF-12 item. This suggests that even independently of physical and mental functioning, ethnicity seems not to affect HRQOL.

A second limitation was the non-random sampling of the study participants. Given the often poor response in migrant groups and the risk that the role of CHWs in recruiting participants in a strictly randomised design conflicts with their role as community health advocates [26], we believed that random sampling was not appropriate for our study. This has most likely not biased the selection of the study population because approximately half of the total number of elderly from each ethnicity, approximately 42.9% Turks, 46.6% Moroccans, and 67.3% Moluccans, who were approached participated in the study.

A third limitation was the unbalanced response rate. The overall response rate of 42.1% was acceptable and comparable with previous research among elderly immigrants (48%) [1]. However, as in the study by Schellingerhout (65.3% Moroccans, 43.6% Turks, Moluccans not reported) [1], the response rate in our study varied strongly across the three ethnic groups (38.1% Moroccans, 79.3% Turks, and 25.4% Moluccans). A non-response analysis showed no selection according to age and gender for Moroccans and Turks. Selection for Moluccans was found regarding age (a lower average age among the non-participants: 63.8 versus 69.7) but not gender. Because our findings are consistent with previous research [1], this, as well as the small number of Moluccans, has not likely influenced the results for Moluccan participants. Another possible risk for bias was that we, due to the limited number of first generation Moluccan immigrants, also included descendants of Moluccan immigrants born and raised in resettlement camps in the Netherlands. We compared Moluccan immigrants with descendants born in the resettlement camps and found that no differences between the two groups, except of course for age, existed in socio-demographic features, health characteristics and HRQOL.

Fourth, not all measuring instruments were available in the appropriate language for the immigrant groups studied, and they were therefore translated. We presented Cronbach’s alpha in the total population and for the three immigrant groups separately for PCS, MCS, D-PAS, C-PAS, and the De Jong Gierveld loneliness scale. These scales showed a good to acceptable reliability in all three groups, with alphas varying from 0.69 to 0.93. We can conclude that these instruments are appropriate for the immigrant groups.

Finally, the self-reported data may have resulted in either report or recall bias. Previous research on HRQOL [6] determined that cross-cultural differences in health perceptions might influence self-reported HRQOL. One might hypothesise that the conceptualisation of health and quality of life depends on cross-cultural differences. Because we found that the PCS and MCS were highly reliable (0.75-0.90), we conclude that the underlying structure of the concept of quality of life is the same for all three immigrant groups.

## Conclusion

Multimorbidity and loneliness, rather than ethnicity, determine the level of HRQOL reported by elderly immigrant populations. Our results suggest that health and prevention programmes meant to improve HRQOL within the ethnic groups do not need to be specific for a particular elderly immigrant population but, rather, should address loneliness and multimorbidity. A potential successful approach to improve HRQOL, for both immigrant and native elderly, could be to screen for developing loneliness and multimorbidity. This could be particularly supportive for Moroccan and Turkish elderly who are most at risk. In addition, interventions addressing loneliness and chronic diseases should be provided as a regular part of usual care. But additional interventions when meant to remove HRQOL differences between the ethnic groups are needed in case of Moroccan and Moluccan elderly. Finally, health literacy deserves attention as immigrants often lack the requisite health literacy skills to maintain health [27]. Therefore culturally sensitive communication programmes are needed for providing immigrant elderly with the health information they need to accessing and making sense of relevant health information.
